# Cognitive Impairment in Temporal Lobe Epilepsy: Alterations in the Basal Forebrain Cholinergic System

**DOI:** 10.1002/brb3.71090

**Published:** 2025-11-19

**Authors:** Beibei Shen, Lina Jiang, Yi Shi, Jiahui Guo, Bofei Chen, Zhiruo Qiu, Shuang Wang, Zhefeng Yuan, Jiajia Fang

**Affiliations:** ^1^ Department of Neurology, the Fourth Affiliated Hospital of School of Medicine, and International School of Medicine, International Institutes of Medicine Zhejiang University Yiwu Zhejiang Province China; ^2^ Department of Radiology, the Fourth Affiliated Hospital of School of Medicine, and International School of Medicine, International Institutes of Medicine Zhejiang University Yiwu Zhejiang Province China; ^3^ Department of Neurology, Epilepsy Center, the Second Affiliated Hospital Zhejiang University School of Medicine Hangzhou Zhejiang Province China; ^4^ Department of Neurology, Children's Hospital Zhejiang University School of Medicine, National Clinical Research Center for Child Health Hangzhou Zhejiang Province China; ^5^ Department of Neurology The First Affiliated Hospital of Huzhou University (Huzhou First People's Hospital) Huzhou Zhejiang Province China

**Keywords:** cognition, fiber projection, basal forebrain cholinergic system, temporal lobe epilepsy

## Abstract

**Objective:**

Temporal Lobe Epilepsy (TLE) often leads to cognitive decline. The Basal Forebrain Cholinergic System (BFCS), essential for memory processes, may play a critical role. This study investigates BFCS alterations, associated white matter tracts, and cognitive correlations in TLE patients.

**Methods:**

We analyzed 100 unilateral TLE patients and 25 healthy controls. Neuropsychological assessments included the Wechsler Memory Scale‐Revised and the Rey Complex Figure Test. Regions of interest (ROIs), such as the hippocampus, amygdala, and dorsolateral prefrontal cortex, were identified using the AAL3 atlas and SPM 8 Anatomy toolbox. BFCS tract integrity was assessed using FSL probabilistic tractography, focusing on FA and MD values.

**Results:**

Both left TLE (LTLE) and right TLE (RTLE) groups displayed significant deficits in memory quotient, verbal/nonverbal memory, and visuospatial working memory, with hippocampal sclerosis (HS) patients showing greater impairment. LTLE patients exhibited extensive BFCS impairment versus controls, with reduced FA (bilateral hippocampus/amygdala, all *p* < 0.01) and elevated MD (bilateral hippocampus *p* < 0.05; bilateral amygdala *p* < 0.01; DLPFC *p* < 0.05). RTLE showed localized damage: decreased FA (left hippocampus/right amygdala, both *p* < 0.05) and increased DLPFC MD (*p* < 0.05). HS intensified BFCS‐right hippocampal and bilateral amygdala damage in TLE. Correlation analyses indicated positive associations between BFCS‐right hippocampal FA and verbal and nonverbal memory, whereas its MD negatively correlated with nonverbal memory decline. BFCS‐right amygdala FA correlated with both memory domains, and BFCS‐DLPFC MD inversely correlated with memory quotient. Elevated MD in the right Ch4 to ipsilateral hippocampal tracts served as a sensitive imaging biomarker for nonverbal memory impairment.

**Conclusion:**

TLE patients exhibit cognitive and visual‐spatial memory deficits, with BFCS tract damage being more pronounced in LTLE and HS patients. The BFCS and its projection fibers demonstrate potential correlations with cognitive function and may be involved in the neural mechanisms of cognitive impairment in TLE.

## Graphical abstract

Temporal lobe epilepsy impairs structural integrity of the BFCS and its projections. Damage to these pathways, especially the right Ch4 to hippocampal tracts, correlates with cognition deficits, identifying BFCS dysfunction as a key mechanism and potential treatment target for cognitive impairment in TLE.

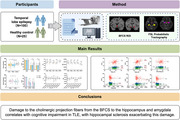

**Abbreviations**
Ach: Acetylcholine
AD: Alzheimer's Disease
Amy: Amygdalae
ASM: Anti‐Seizure Medicines
BFCS: Basal Forebrain Cholinergic System
DBB: Diagonal Band of Broca
DLPFC: Dorsal Prefrontal Cortex
DTI: Diffusion Tensor Imaging
EEG: Electroencephalography
EPI: Echo‐planar Imaging
FA: Fractional Anisotropy
FC: Functional Connectivity
FDT: FMRIB's Diffusion Toolbox
FSL: FMRIB Software Library
GTCS: Generalized Tonic‐Clonic Seizure
hDBB: Horizontal Limb of DBB
HS: Hippocampal Sclerosis
ILAE: International League Against Epilepsy
LTLE: Left Temporal Lobe Epilepsy
MD: Mean Diffusivity
MQ: Memory Quotient
MRI: Magnetic Resonance Imaging
MS: Medial Septum, Ch1
NBM: Nucleus Basalis of Meynert, Ch4
ROCFT: Rey‐Osterrieth Complex Figure Test
ROI: Region of Interest
RTLE: Right Temporal Lobe Epilepsy
SD: Standard Deviation
SEEG: Stereo‐Electroencephalography
TE: Echo Time
TI: Inversion Time
TLE: Temporal Lobe Epilepsy
TR: Repetition Time
vDBB: Vertical Limb of DBB
WMS‐RC: Wechsler Memory Scale‐Revised

## 1 Introduction

Temporal lobe epilepsy (TLE), the most common adult epilepsy syndrome, is frequently accompanied by progressive cognitive decline, particularly in memory domains, significantly impairing quality of life (Chauvière 2020; Chauvière et al. 2009). While current anti‐seizure therapies effectively control seizures, they may paradoxically worsen cognitive deficits such as memory impairment and executive dysfunction, forcing clinicians to navigate a precarious balance between seizure suppression and functional preservation (Sarkis et al. 2018; Keezer et al. 2016).

Cognitive dysfunction in TLE arises from multifactorial pathologies, including hippocampal damage, neural network disconnection, and metabolic dysregulation (Englot et al. 2020; Laurent et al. 2020). Neuroimaging evidence demonstrates a significant inverse correlation between hippocampal volume reduction and cognitive deterioration, particularly in refractory TLE with hippocampal sclerosis (Ramm et al. 2020; Agudelo Valencia et al. 2021). White matter tract disruption directly impacts verbal memory (Gleichgerrcht et al. 2021), while metabolic disturbances extend beyond temporal regions to involve the hippocampus, amygdala, and cerebellum, impairing memory encoding and information integration (Guo et al. 2022). Despite progress in understanding TLE‐related cognitive impairment, clinical translation remains hindered by the lack of early biomarkers and neuroprotective therapies, alongside overreliance on seizure metrics. Integrating brain network biomarkers with multidimensional cognitive assessments could enable dual optimization of seizure control and neural preservation, improving long‐term outcomes.

Central to cognitive regulation is the basal forebrain cholinergic system (BFCS), the primary source of cortical and limbic cholinergic input (Ananth et al. 2023). Recent research has demonstrated that the BFCS is implicated in cognitive alterations observed in various neurodegenerative disorders, including Alzheimer's disease (AD) and Parkinson's disease (PD) (Liu et al. 2023; Pereira et al. 2020). The BFCS can be delineated into four distinct subdivisions: Ch1‐2, Ch3, and Ch4 (Raghanti et al. 2011; Woolf 1991). The Ch1‐2 subdivision encompasses the medial septum (MS) and the vertical limb of the diagonal band of Broca (vDBB). The Ch3 subdivision is characterized by the horizontal limb of the DBB (hDBB). The Ch4 subdivision comprises the nucleus basalis of Meynert (NBM), wherein NBM neurons extend cholinergic fibers to the cerebral cortex, including regions such as the prefrontal cortex, as well as to the amygdala (Ananth et al. 2023; Crowley et al. 2024). Additionally, cholinergic neurons within the MS predominantly project fibers via the fimbria, critically regulating cortical activation and short‐term memory consolidation. Reduced efficiency of cholinergic connectivity between the BFCS and corticolimbic networks represents a transdiagnostic neural mechanism underpinning cognitive decline in both AD and epilepsy (Zeng et al. 2022; Ballinger et al. 2016).

Our previous investigations have demonstrated a positive correlation between cognitive impairment in TLE patients and pathological alterations in the MS‐hippocampal pathway (Chen et al. 2023). Consequently, the present study seeks to explore the alterations in the BFCS and its related white matter tracts in TLE patients, as well as their relationship with cognitive function. This research aims to establish a foundation for enhancing cognitive health in epilepsy patients by studying the effects of BFCS and its white matter connections on cognition, enabling more targeted interventions for TLE‐related cognitive issues.

## 2 Method

### 2.1 Participants

This study encompassed a retrospective review of 50 patients with left temporal lobe epilepsy (LTLE), 50 patients with right temporal lobe epilepsy (RTLE), and 25 healthy volunteers, all of whom were under the care of the Epilepsy Center at the Second Affiliated Hospital of Zhejiang University between October 2018 and July 2024. Informed written consent was obtained from each participant. This study was approved by the Ethics Committee of the Second Affiliated Hospital, Zhejiang University School of Medicine (approval no. 2014151). All subjects underwent Magnetic Resonance Imaging (MRI) utilizing a 3.0 Tesla MRI scanner. Diagnostic assessments were conducted by a minimum of two experienced epilepsy specialists. These assessments were based on a comprehensive evaluation that included the patients' clinical presentations, long‐term scalp video electroencephalography (EEG), MRI scans, and neuropsychological examination. The MRI scans revealed hippocampal atrophy and signal abnormalities, with some cases pathologically confirmed as hippocampal sclerosis. A subset of patients received a diagnosis through stereo‐electroencephalography (SEEG), a more invasive procedure that involves the placement of depth electrodes directly into the brain tissue.

Inclusion Criteria: (1) The types of seizures and clinical symptoms must align with the diagnostic and classification standards established by the International League Against Epilepsy (ILAE) in 2017 (Fisher et al. 2017), with TLE diagnoses confirmed by EEG analysis. (2) Age between 15 and 60 years. (3) Right‐handedness. (4) Stability to undergo imaging and cognitive assessments. (5) Native Chinese speaker.

Exclusion Criteria: (1) A history of or current craniocerebral trauma, infectious diseases (e.g., encephalitis, meningitis), vascular diseases (e.g., malformations, strokes), tumors, developmental malformations, or other central nervous system disorders. (2) Multiple epileptic foci. (3) Mental disorders or low educational levels that preclude cooperation with imaging and cognitive assessments. (4) Long‐term medication use, excluding ASMs. (5) Lactation or pregnancy.

Healthy volunteers were selected with the following requirements: (1) Gender, age, and language matched to the patient groups. (2) Right‐handedness. (3) No history of neurological or psychiatric illness. (4) Normal findings on head MRI. (5) No mental issues or educational limitations that would hinder participation in imaging and cognitive evaluations.

### 2.2 Neuropsychological Assessments

Neuropsychological assessments, including the Verbal Paired Associates and Logical Memory subtests from the Wechsler Memory Scale‐Revised (WMS‐RC), were conducted on patients and healthy controls to evaluate verbal memory (Ji et al. 2018). Nonverbal memory was assessed using the recognition and visual reproduction subtests (Vythilingam et al. 2004). Raw scores from immediate recall tasks were calculated and converted into standard scores, then aggregated into a total score to determine an age‐adjusted Memory Quotient (MQ). The Rey‐Osterrieth Complex Figure Test (ROCFT) assessed visual‐spatial memory, using copy, instant, and delayed recall scores to measure performance (Jamus et al. 2023).

### 2.3 Imaging and Data Acquisition

All participants underwent examination at the Second Affiliated Hospital of Zhejiang University School of Medicine utilizing a 3.0 Tesla Discovery MR750 scanner (GE Healthcare) equipped with an 8‐channel cranial coil. The scanning protocol comprised two sequences, detailed with their respective parameters as follows:

1. The Sagittal T1‐Weighted Three‐Dimensional Brain Volume Image (3DT1/3D BRAVO) protocol is characterized by a repetition time (TR) of 8.2 ms, an echo time (TE) of 3.2 ms, an inversion time (TI) of 450 ms, and a flip angle of 12°. The matrix size is 256 × 256, with a sagittal plane resolution of 0.47 × 0.47 mm^2^, a slice thickness of 1 mm, and a total of 206 slices. This protocol ensures high‐resolution structural imaging of the brain, thereby facilitating a detailed analysis of anatomical structures.
2. Diffusion Tensor Imaging (DTI) was conducted using a spin‐echo echo‐planar imaging (EPI) sequence with the following parameters: TR/TE = 8000/80.8 ms, FA = 90°, matrix size of 128 × 128, slice thickness = 2 mm across 67 slices. The maximum *b*‐value utilized was 1000 s/mm^2^ across 30 non‐collinear directions, with 5 volumes acquired without diffusion weighting (*b*‐value = 0 s/mm^2^).

### 2.4 Image Analysis

#### 2.4.1 Image Processing and Analysis

The FMRIB's Diffusion Toolbox (FDT) from the FMRIB Software Library (FSL) software (version 5.0.11, http://fsl.fmrib.ox.ac.uk/fsl) processes diffusion data. Initially, MRIcron converts each subject's images to four‐dimensional NIFTI data, and the diffusion images are verified. Susceptibility distortion correction is applied, and BET is used for brain extraction on the corrected B0 output to generate a brain mask. Eddy Correct is used for eddy current correction, after which diffusion tensor images are fitted to the corrected data.

#### 2.4.2 Region of Interest (ROI) Division and Calculation of BFCS Volume

The MNI152 standard brain was initially aligned with each subject's T1 image, which was then aligned with their DTI image. Regions of interest, including the hippocampus, amygdalae, and dorsolateral prefrontal cortex, were extracted from the MNI152 space using the AAL3 atlas, based on previous studies (Wang et al. 2020). ROIs like BFCS and its subregions were extracted from MNI152 using the SPM8 Anatomy toolbox in MATLAB 2018a (Zeng et al. 2022; Butler et al. 2014). Their volumes were calculated using the fslstats method in FSL software.

#### 2.4.3 Probabilistic Tractography

In T1‐weighted images, ROIs in the BFCS are aligned with the diffusion space. Using FSL's probabilistic tractography, we trace fiber connections from the BFCS to the amygdala, hippocampus, and dorsolateral prefrontal cortex. BFCS subregions act as seed masks, while the hippocampus, amygdala, and dorsolateral prefrontal cortex are used as waypoint and termination masks for fiber tracking. The fslstats tool in FSL is used to compute the fractional anisotropy (FA) and mean diffusivity (MD) values of BFCS‐related fiber tracts for each subject.

### 2.5 Statistics

**FIGURE 1:**
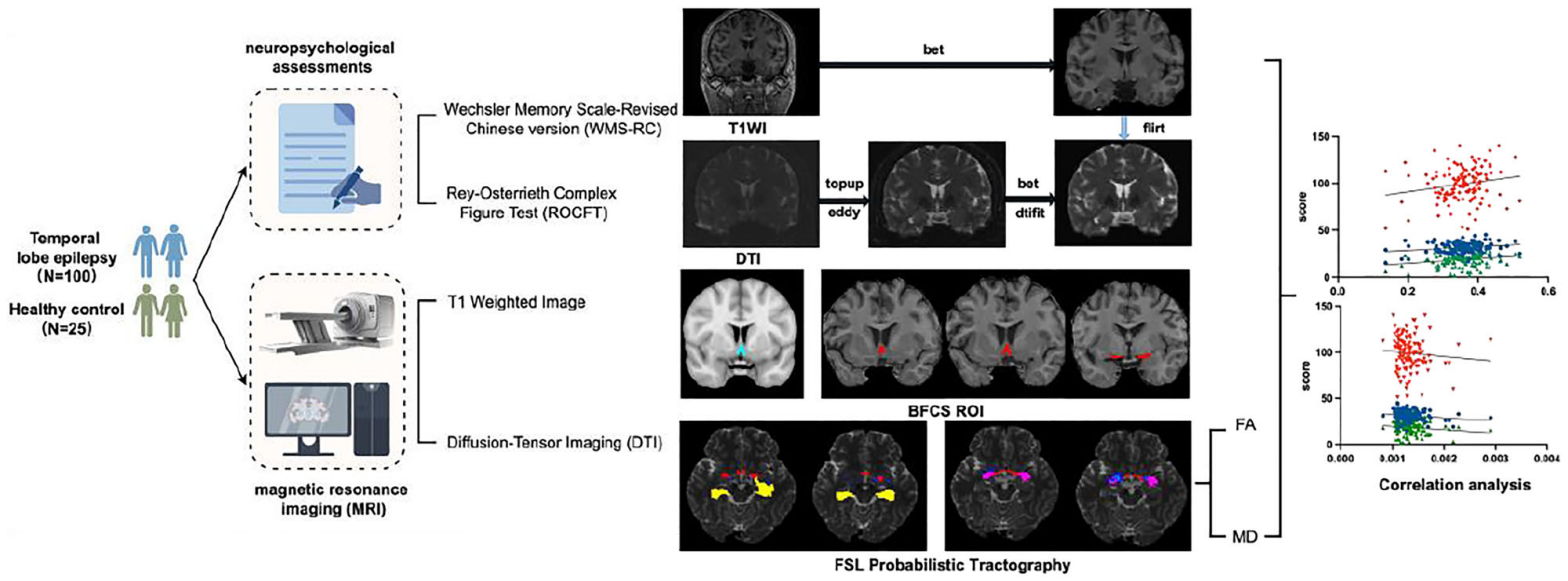
Clinical trial flow diagram. A comprehensive neuropsychological evaluation and MRI scans were administered to participants diagnosed with TLE as well as to healthy controls. After the acquisition of T1‐weighted and DTI data, ROIs were delineated, and fiber tractography was executed. DTI metrics were extracted for comparative analysis, and correlation analyses were performed to assess relationships between variables.

Data analysis was conducted using SPSS 29.0.1. The Kolmogorov‐Smirnov test checked data normality. Data are reported as the mean ± standard deviation (SD). Independent samples *t*‐tests were performed for comparisons between two groups. For multiple group comparisons, one‐way analysis of variance (ANOVA) with Tukey's post‐hoc test was adopted, with Bonferroni correction applied to *p*‐values. Categorical data were shown as counts and percentages, with group comparisons made using the chi‐square test. Variable relationships were assessed using Spearman correlation followed by partial correlation analysis, with FDR correction applied to correlation *p*‐values. A two‐tailed *p‐*value < 0.05 was considered statistically significant. Figure 1 illustrates the clinical trial methodology employed in this study.

## 3 Results

### 3.1 Clinical Characteristics and Cognitive Results

Table 1 provides a detailed overview of the demographic characteristics of all participants. One‐way ANOVA confirmed no significant differences in age and gender across the three groups, whereas a significant difference in educational attainment was observed (*F* = 13.041, *p* < 0.001). One‐way ANOVA with Tukey test revealed that both LTLE and RTLE groups showed significantly lower educational attainment compared to the control group (LTLE: 13.04 ± 3.45, control group: 16.04 ± 1.87, *p2* < 0.001; RTLE: 12.78 ± 3.10, control group: 16.04 ± 1.87, *p3* < 0.001), while no statistically significant difference was observed between the two patient groups themselves. All reported *p*‐values have been Bonferroni‐corrected. Independent samples *t*‐tests revealed no significant differences between the LTLE and RTLE groups in the incidence of GTCS, refractory epilepsy status, or HS prevalence.

**TABLE 1:** Demographic and clinical data.

Clinical variables	LTLE(*N* = 50)	RTLE(*N* = 50)	Control(*N* = 25)	*p‐*value	Post hoc tests *p‐*value		
					*p1* value	*p2* value	*p3* value
Age (years)	32.98 ± 9.77	32.04 ± 10.40	31.36 ± 8.72	0.733	0.733	0.424	0.625
Education (years)	13.04 ± 3.45	12.78 ± 3.10	16.40 ± 1.87	< 0.001	0.688	< 0.001	< 0.001
Sex (N, %)							
Male	26(52.0%)	19(38.0%)	10(40.0%)	0.334	0.159	0.327	0.867
Female	24(48.0%)	31(62.0%)	15(60.0%)				
Intractable epilepsy (N,%)							
yes	9(18.4%)	15(30.6%)	N/A		0.195		
no	40(81.6%)	34(69.4%)	N/A				
GTCS (N,%)							
yes	27(57.4%)	35(71.4%)	N/A		0.152		
no	20(42.6%)	14(28.6%)	N/A				
HS (N,%)	32(64.0%)	31(62.0%)	N/A		0.836		

*Note*: All data are presented as mean ± SD. The *p*‐value denotes ANOVA comparisons among LTLE, RTLE, and control groups, while the *p1* value, *p2* value, and *p3* value represent Bonferroni‐corrected pairwise comparisons for LTLE vs. RTLE, LTLE vs. control, and RTLE vs. control, respectively.

Abbreviations: Control, healthy control group; GTCS, Generalized Tonic‐Clonic Seizure; HS, hippocampal sclerosis; LTLE, left temporal lobe epilepsy; RTLE, right temporal lobe epilepsy.

**FIGURE 2:**
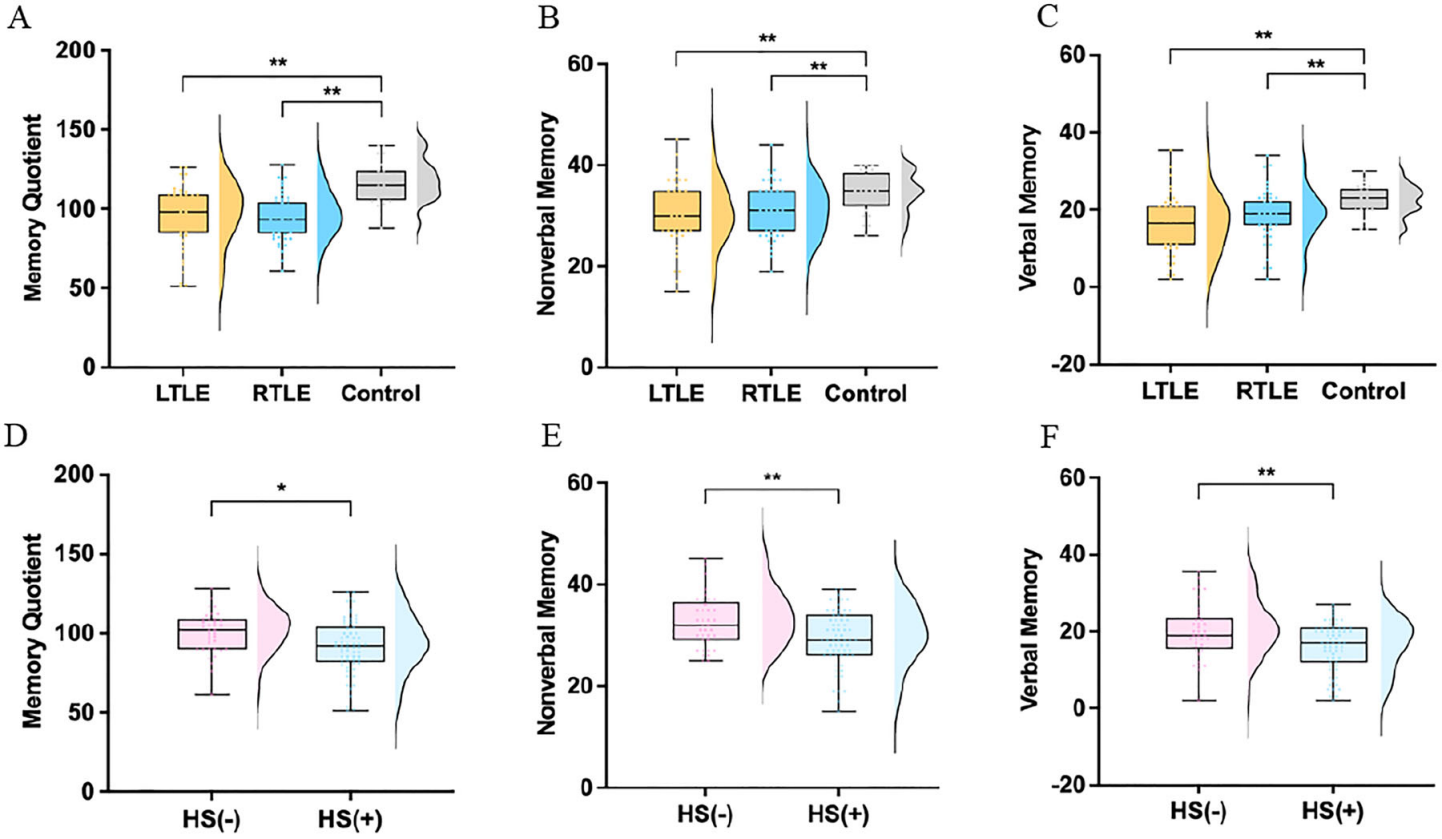
Comparative analysis of cognition across diverse groups. (A‐C) memory quotient (A), nonverbal (B), and verbal memory (C) were compared between healthy controls and TLE patients; (D‐F) Memory quotient (D), nonverbal (E), and verbal memory (F) were compared between HS(‐) and HS(+) patients. The boxplot whiskers extend to the most extreme data points within 1.5 × IQR from the quartiles. LTLE: left temporal lobe epilepsy; RTLE: right temporal lobe epilepsy; Control: healthy control group; HS (‐): TLE without hippocampal sclerosis; HS (+): TLE with hippocampal sclerosis; *p < 0.05, **p < 0.01.

**FIGURE 3:**
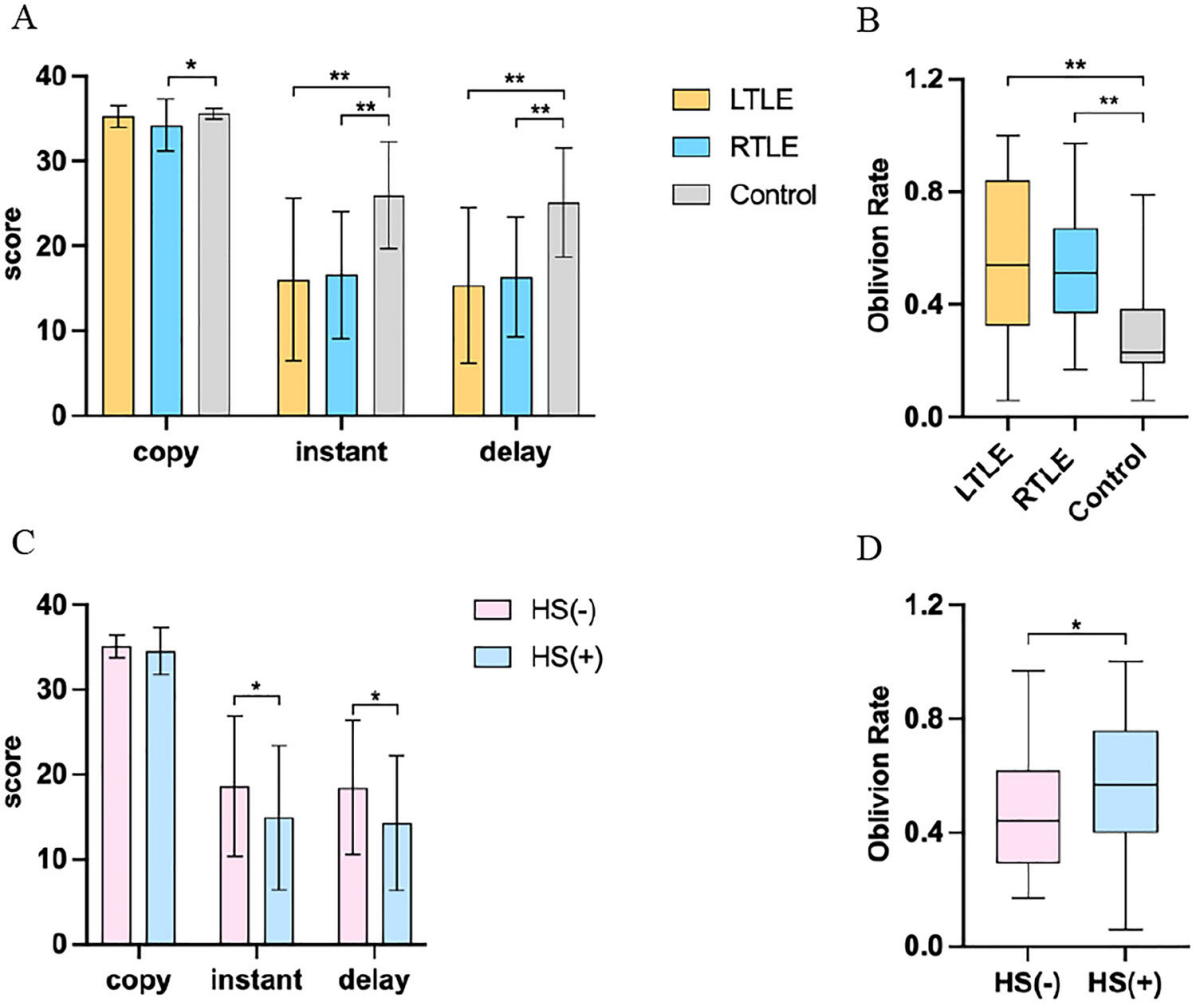
Comparison of visual‐spatial memory across diverse groups. (A) Visual‐spatial memory was compared between healthy controls and TLE patients; (B) Oblivion rate was compared between healthy controls and TLE patients. (C) Visual‐spatial memory was compared between HS(‐) and HS(+) patients; (D) Oblivion rate was compared between HS(‐) and HS(+) patients. The boxplot whiskers extend to the most extreme data points within 1.5 × IQR from the quartiles. copy: copy score; instant: instant visual‐spatial memory score; delay: delayed visual‐spatial memory score; LTLE: left temporal lobe epilepsy; RTLE: right temporal lobe epilepsy; Control: healthy control group; HS (‐): TLE without hippocampal sclerosis; HS (+): TLE with hippocampal sclerosis; oblivion rate=(copy‐instant) / copy; *p < 0.05, **p < 0.01.

One‐way ANOVA revealed significant differences among the three groups in cognitive level and visual‐spatial memory, with specific manifestations in Memory Quotient (MQ) (*F* = 21.334, *p* < 0.001), nonverbal memory (*F* = 7.138, *p* = 0.001), verbal memory (*F* = 10.244, *p* < 0.001), copy (*F* = 4.565, *p* = 0.012), instant (*F* = 13.907, *p* < 0.001), and delayed recall score (*F* = 14.116, *p* < 0.001). One‐way ANOVA with Tukey test demonstrated that both LTLE and RTLE groups exhibited significant differences in memory quotient (LTLE: 95.26 ± 18.70, control: 117.80 ± 12.89, *p <* 0.01; RTLE: 94.10 ± 13.95, control: 117.80 ± 12.89, *p <* 0.01), verbal memory (LTLE: 30.18 ± 6.05, control: 35.00 ± 4.03, *p <* 0.01; RTLE: 31.06 ± 5.07, control: 35.00 ± 4.03, *p <* 0.01), nonverbal memory (LTLE: 16.15 ± 7.27, control: 23.12 ± 3.46, *p <* 0.01; RTLE: 18.7 ± 6.31, control: 23.12 ± 3.46, *p <* 0.01) (Figure 2A‐C), instant (LTLE: 16.06 ± 9.61, control: 26.00 ± 6.33, *p <* 0.01, RTLE: 16.62 ± 7.49, control: 26.00 ± 6.33, *p <* 0.01) and delay visual‐spatial memory (LTLE: 15.36 ± 9.18, control: 25.13 ± 6.43, *p <* 0.01, RTLE: 16.34 ± 7.03, control: 25.13 ± 6.43, *p <* 0.01) when compared to the control group (Figure 3A). All reported *p‐*values were derived after Bonferroni correction. Additionally, chi‐square tests identified higher oblivion rates in LTLE and RTLE relative to control (LTLE: 0.57 ± 0.26, control: 0.30 ± 0.18, *p <* 0.01, RTLE: 0.53 ± 0.19, control: 0.30 ± 0.18, *p <* 0.01) (Figure 3B). Notably, despite significant impairments across multiple cognitive domains in both TLE groups, no statistically significant differences were observed between LTLE and RTLE in MQ, verbal memory, or nonverbal memory performance (Figure 2A‐C). These findings suggest that cognitive deficits associated with unilateral TLE may predominantly manifest as disruptions in spatial information processing.

To examine whether hippocampal sclerosis independently contributes to cognitive impairment in individuals with TLE, we categorized patients according to the presence of hippocampal sclerosis and subsequently analyzed their cognitive outcomes. Independent two‐sample *t*‐tests revealed significant cognitive differences between HS(‐) and HS(+) groups. The HS(‐) group showed higher memory quotients (HS(‐): 99.95 ± 13.34, HS(+): 91.59 ± 17.36, *p <* 0.05), superior verbal memory (HS(‐): 19.99 ± 7.21, HS(+): 15.94 ± 6.29, *p <* 0.01), better nonverbal memory (HS(‐): 32.68 ± 5.12, HS(+): 29.41 ± 5.51, *p <* 0.01) (Figure 2D‐F), enhanced instant visual‐spatial memory (HS(‐): 18.68 ± 8.29, HS(+): 14.95 ± 8.51, *p <* 0.05), and improved delayed visual‐spatial memory (HS(‐): 18.47 ± 7.88, HS(+): 14.29 ± 7.96, *p <* 0.05) (Figure 3C). Additionally, chi‐square testing demonstrated a significantly increased oblivion rate in HS(+) patients (HS(‐): 0.47 ± 0.22, HS(+): 0.59 ± 0.22, *p <* 0.05) (Figure 3D). These findings indicate that TLE patients with HS exhibit more pronounced impairments in global cognitive function and visual‐spatial memory compared to TLE without HS, suggesting that HS not only serves as an anatomical biomarker of cognitive dysfunction in TLE but may also exacerbate cognitive decline through disruption of neural circuits critical for memory encoding and storage.

### 3.2 The Volumes and Fiber Connections of BFCS

Our comparative analysis revealed significant laterality differences in BFCS projection network fiber connectivity through comparative analysis of TLE patients. One‐way ANOVA revealed statistically significant differences in BFCS‐related fiber connectivity parameters across three groups, with significant variations observed in the FA values of BFCS projections to bilateral hippocampus (Lhipp: *F =* 5.280, *p =* 0.006, Rhipp: *F =* 7.749, *p =* 0.001) and bilateral amygdala (Lamy: *F =* 5.190, *p =* 0.007, RAmy: *F =* 7.382, *p =* 0.001), as well as in the MD values of BFCS projections to the right hippocampus (*F =* 5.631, *p =* 0.005), bilateral amygdala (Lamy: *F =* 5.727, *p =* 0.004, RAmy: *F =* 6.549, *p =* 0.002), and DLPFC (*F =* 3.397, *p =* 0.037).

One‐way ANOVA with Tukey's post‐hoc test demonstrated that compared to healthy controls, individuals with LTLE exhibited significantly reduced FA values (Lhipp: *p <* 0.01, Rhipp: *p <* 0.01; LAmy: *p <* 0.01, RAmy: *p <* 0.01) and increased MD values (Lhipp: *p <* 0.05, Rhipp: *p <* 0.05; LAmy: *p <* 0.01, RAmy: *p <* 0.01) in the BFCS fiber connections to both the bilateral hippocampus and bilateral amygdalae. Furthermore, the MD values of the BFCS to DLPFC fiber junction were significantly elevated in the LTLE group compared to the control group (*p <* 0.05). The RTLE group exhibited notably lower FA values in fiber connections to the left hippocampus (*p <* 0.05) and right amygdala (*p <* 0.05) and higher MD values to the DLPFC (*p <* 0.05), compared to the control group. All reported *p‐*values were derived after Bonferroni correction. Paired *t*‐tests within controls revealed no anatomical asymmetry in BFCS‐related fiber connections.

In comparison to the RTLE group, the FA value of the BFCS to the fiber junction of the right hippocampus was significantly reduced, while the MD value was significantly elevated in the LTLE group (*p <* 0.05, *p <* 0.01). Additionally, the LTLE group exhibited a significant increase in the MD value in the bilateral amygdala (*p <* 0.05 for both comparisons). This prompted us to examine the consistency of alterations within the projection fibers of the BFCS. Consequently, we conducted a comprehensive analysis of the projection fibers, with particular attention to the subregions of the BFCS. Our study aimed to ascertain whether the observed changes in fiber projections were uniform across different segments of the BFCS, potentially offering deeper insights into the neurological mechanisms underlying TLE and its cognitive ramifications.

**FIGURE 4:**
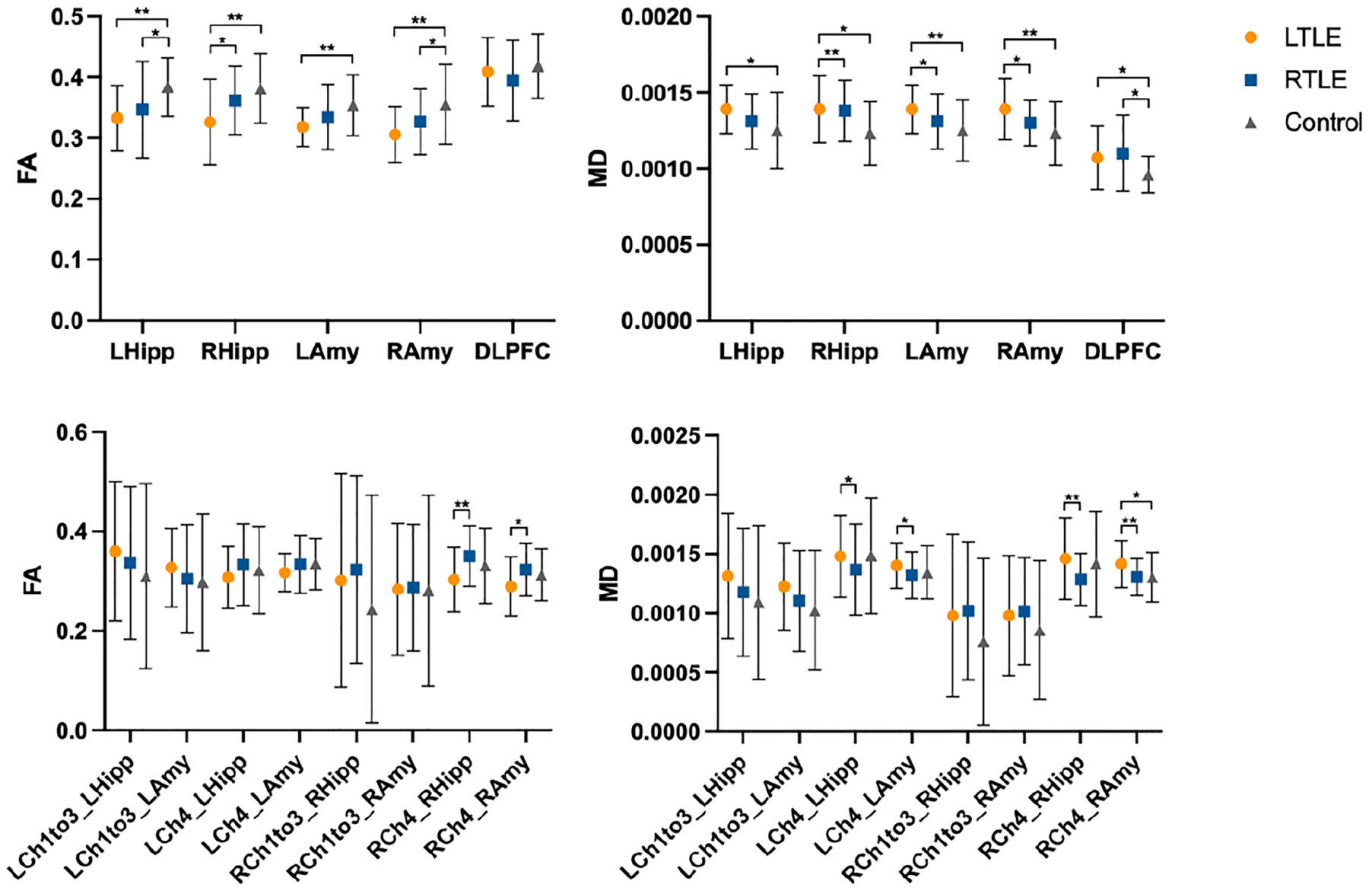
White matter fiber connections and associated parameter changes of BFCS to amygdala, hippocampus, and dorsolateral prefrontal cortex. (A, B) Comparison of the FA (A) and MD (B) in the BFCS connections to the hippocampus, amygdalae, and prefrontal cortex between healthy controls and TLE patients; (C, D) Comparison of the same measures (FA and MD) in BFCS subregions (Ch1‐3, Ch4) among the three groups. Hipp: hippocampus; Amy: amygdala; DLPFC: dorsal prefrontal cortex; FA: fractional anisotropy; MD: mean diffusivity; Ch1to3: BFCS subregion Ch1‐3 nuclei; Ch4: BFCS subregion Ch4 nucleus; LTLE: left temporal lobe epilepsy; RTLE: right temporal lobe epilepsy; Control: healthy control group; L: left side; R: right side; *p < 0.05, **p < 0.01.

We found that the three groups also demonstrated significant differences in certain fiber projections of BFCS‐related subregions, specifically manifested in the FA values of the right Ch4 to the right hippocampus (*F =* 6.549, *p =* 0.002) and right amygdala (*F =* 4.765, *p =* 0.010), as well as the MD values to the right amygdala (*F =* 5.394, *p =* 0.006). A Bonferroni correction was applied to all reported *p*‐values. Significantly, in comparison to the RTLE group, the LTLE group exhibited more pronounced impairment in the fiber connections from bilateral Ch4 to the ipsilateral hippocampus and ipsilateral amygdala. This was evidenced by a reduction in FA values (RCh4_RHipp: *p <* 0.01, RCh4_RAmy: *p <* 0.05) and an elevation in MD values (RCh4_RHipp: *p <* 0.01, RCh4_RAmy: *p <* 0.01), as illustrated in Figure 4. However, the groups showed no significant differences in BFCS, L/RCh1‐3, and L/RCh4 volumes (Table 2).

**TABLE 2:** Comparison of BFCS volumes across different groups.

Clinical variables	LTLE(*N* = 50)	RTLE(*N* = 50)	Control(*N* = 25)	*p*‐value	Post hoc tests *p‐*value		
					LTLE vs. RTLE	LTLE vs. Control	RTLE vs. Control
BFCS_Voxel	446.74 ± 47.73	437.92 ± 48.97	436.04 ± 76.51	0.143	0.107	0.525	0.104
LCh1‐3_Voxel	97.16 ± 16.06	92.88 ± 18.45	98.72 ± 22.04	0.741	0.424	0.770	0.800
LCh4_Voxel	132.20 ± 22.47	127.04 ± 18.06	128.52 ± 24.09	0.868	0.783	0.621	0.686
RCh1‐3_Voxel	89.32 ± 16.87	88.42 ± 13.18	89.72 ± 27.41	0.804	0.546	0.997	0.624
RCh4_Voxel	132.30 ± 20.27	129.58 ± 19.72	132.28 ± 30.49	0.753	0.512	0.570	0.787

Abbreviations: BFCS, Basal Forebrain Cholinergic System; Ch1‐3, BFCS subregion Ch1‐3 nuclei; Ch4, BFCS subregion Ch4 nucleus; Control, healthy control group; L, left; LTLE, left temporal lobe epilepsy; R, right; RTLE, right temporal lobe epilepsy.

**FIGURE 5:**
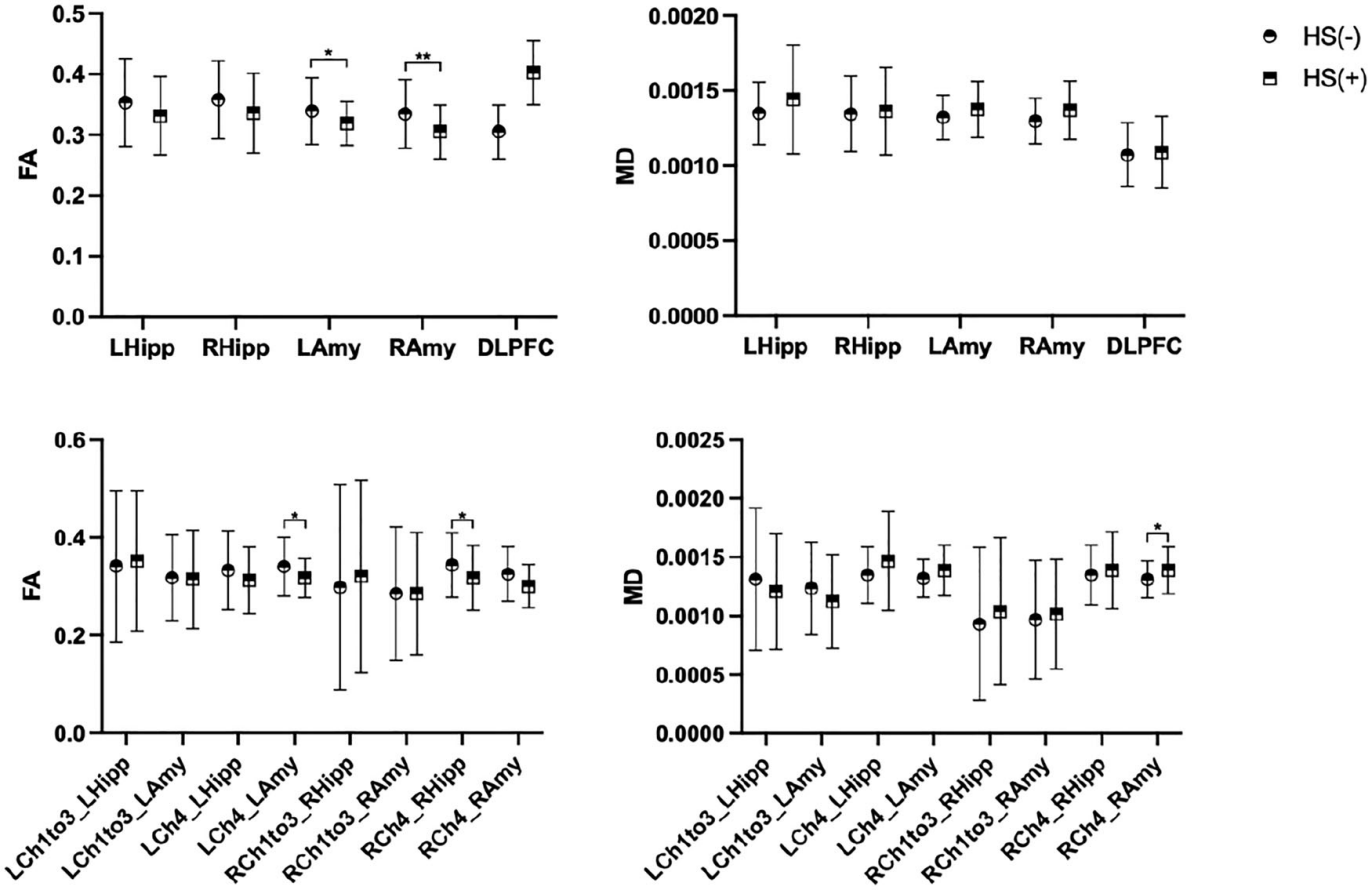
White matter fiber connections and associated parameter changes of BFCS subregions to bilateral amygdalae and hippocampus across different groups. (A, B) Comparison of the FA (A) and MD (B) in the BFCS connections to the hippocampus, amygdalae, and prefrontal cortex in TLE patients with/without HS; (C, D) Comparison of the same measures (FA and MD) in BFCS subregions (Ch1‐3, Ch4) between the two groups. LHipp: left hippocampus; RHipp: right hippocampus; LAmy: left amygdala; RAmy: right amygdala; DLPFC: dorsal prefrontal cortex; FA: fractional anisotropy; MD: mean diffusivity; Ch1to3: BFCS subregion Ch1‐3 nuclei; Ch4: BFCS subregion Ch4 nucleus; HS (‐): TLE without hippocampal sclerosis; HS (+): TLE with hippocampal sclerosis; L: left side; R: right side; *p < 0.05, **p < 0.01.

Independent two‐sample *t*‐tests revealed that compared to the HS (‐) group, the HS (+) group demonstrated a significant reduction in FA values for BFCS fiber connections to both amygdalae (Lamy: *p <* 0.05, Ramy: *p <* 0.01). However, no statistically significant differences were observed in FA and MD values between the two groups concerning connections to the bilateral hippocampus. These findings suggest that TLE patients with HS exhibit more pronounced damage to BFCS fiber connections to the bilateral amygdalae (Figure 5).

When compared to the HS (‐) group, the left Ch4 region exhibited a statistically significant reduction in FA towards the ipsilateral amygdala (*p <* 0.05), whereas the right Ch4 region showed a significant decrease in FA towards the ipsilateral hippocampus (*p <* 0.05) and an increase in MD towards the ipsilateral amygdala (*p <* 0.05) in the HS (+) group. These results suggest that the fiber connections from the left Ch4 to the ipsilateral amygdala, as well as those from the right Ch4 to both the ipsilateral hippocampus and amygdala, are more severely compromised in patients with TLE and hippocampal sclerosis (HS) compared to those without HS. Consistent with the previous findings, there were no significant differences in the volumes of BFCS, L/RCh1‐3, and L/RCh4 between the two groups as presented in Table 3.

**TABLE 3:** Comparison of BFCS volumes in TLE with and without HS.

Clinical variables	HS(‐)(*N* = 37)	HS(+)(*N* = 63)	p value
BFCS_Voxel	442.38 ± 43.43	442.30 ± 51.30	0.994
LCh1‐3_Voxel	95.54 ± 16.66	94.71 ± 17.85	0.819
LCh4_Voxel	127.35 ± 17.93	130.95 ± 21.82	0.398
RCh1‐3_Voxel	88.73 ± 12.67	88.95 ± 16.41	0.944
RCh4_Voxel	130.76 ± 20.01	131.05 ± 20.06	0.944

Abbreviations: BFCS, Basal Forebrain Cholinergic System; Ch1‐3, BFCS subregion Ch1‐3 nuclei; Ch4, BFCS subregion Ch4 nucleus; HS (+), TLE with hippocampal sclerosis; HS (−), TLE without hippocampal sclerosis; L, left; R, right.

### 3.3 Association Between BFCS‐Associated Fiber Connections and Cognition

To maximize statistical power, all TLE patients were analyzed collectively without subgroup stratification by laterality or hippocampal sclerosis status, revealing several nominal associations between BFCS connectivity metrics and cognitive functions.

**FIGURE 6:**
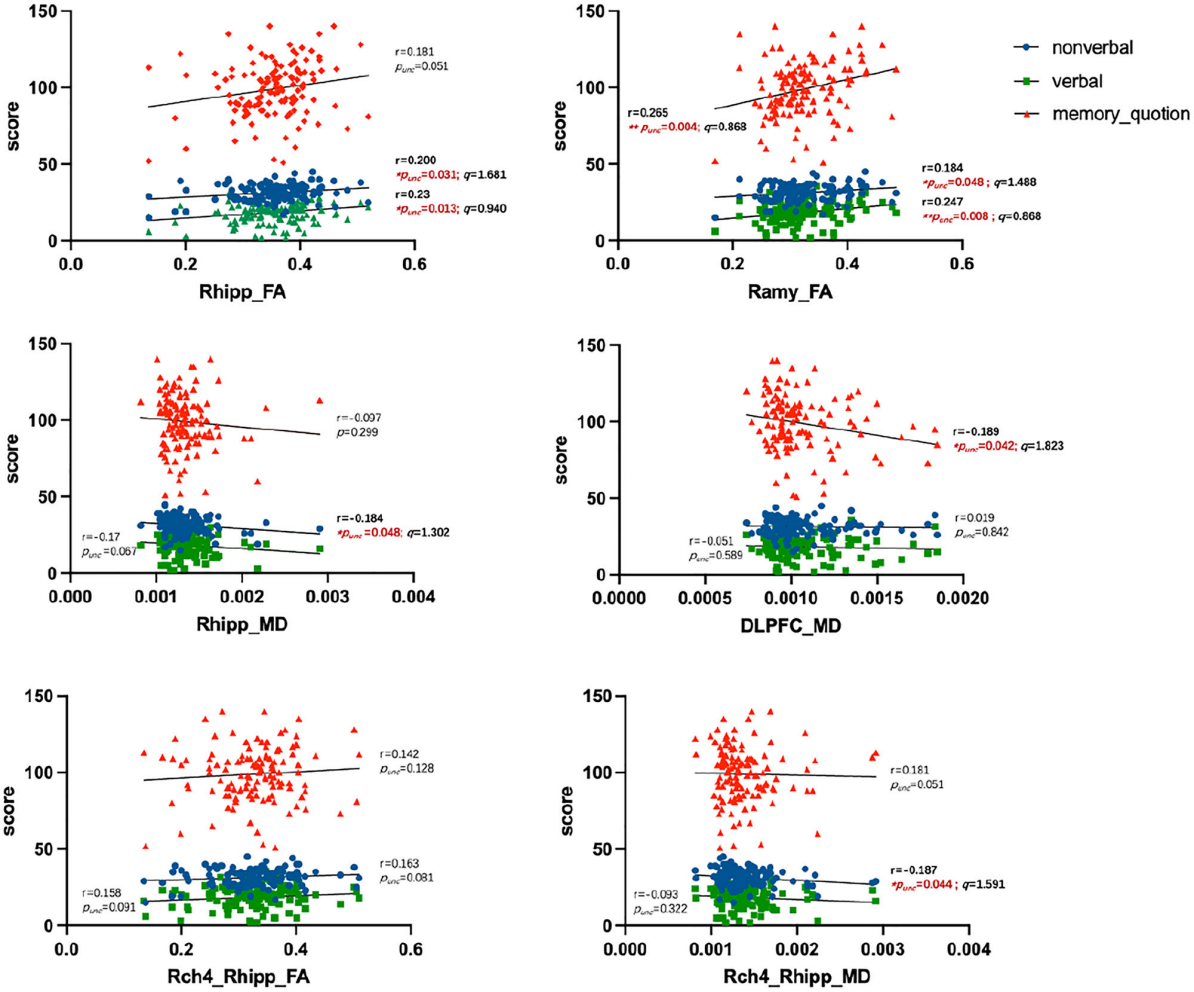
Correlation between changes in BFCS‐related white matter fiber connections and cognitive function. (A, C) Correlation analysis of FA (A) and MD (C) in the fiber connections from the BFCS to the right hippocampus with nonverbal memory, verbal memory, and memory quotient; (B) Correlation analysis of FA in the fiber connections from the BFCS to the right amygdala with nonverbal memory, verbal memory, and memory quotient; (D) Correlation analysis of MD in the fiber connections from the BFCS to the dorsal prefrontal cortex with nonverbal memory, verbal memory, and memory quotient; (E, F) Correlation analysis of FA (E) and MD (F) in the fiber connections from the right Ch4 subregion to the right hippocampus with nonverbal memory, verbal memory, and memory quotient. The correlation coefficients (r values) and *p* values are shown in the figures. hipp: hippocampus; amy: amygdala; DLPFC: dorsal prefrontal cortex; FA: fractional anisotropy; MD: mean diffusivity; ch4: BFCS subregion Ch4 nucleus; L: left side; R: right side; *p_unc_* = uncorrected *p*‐values; *q* = FDR‐corrected *p*‐values, *p_unc_ < 0.05, **p_unc_ < 0.01; *q < 0.05, **q < 0.01.

Pearson and Spearman correlation analyses demonstrated that BFCS‐right hippocampal fiber FA values positively correlated with nonverbal memory (r = 0.200, *p_unc_* = 0.031; *r =* 0.184, *p_unc_* = 0.048, Figure 6A) and verbal memory (*r =* 0.230, *p_unc_* = 0.013; *r =* 0.247, *p_unc_* = 0.008, Figure 6A), whereas MD values of this pathway showed negative correlations with nonverbal memory (*r =* ‐0.184, *p_unc_* = 0.048, Figure 6C). BFCS‐right amygdala FA values similarly exhibited positive correlations with nonverbal memory (*r =* 0.184, *p_unc_* = 0.048, Figure 6B) and verbal memory (*r =* 0.247, *p_unc_* = 0.008, Figure 6B). Concurrently, BFCS to dorsolateral prefrontal cortex fiber MD values negatively correlated with memory quotient (*r =* ‐0.189, *p_unc_* = 0.042, Figure 6D). Notably, elevated MD of right Ch4 to ipsilateral hippocampal tracts demonstrated negative correlations with nonverbal memory decline (*r =* ‐0.187, *p_unc_* = 0.044, Figure 6F). These results indicate that microstructural disruptions in BFCS networks, particularly affecting right Ch4 to hippocampal circuitry, may constitute an underlying substrate for cognitive dysfunction in TLE. Though nonsignificant after FDR correction (all *q* > 0.05, potentially reflecting statistical constraints, Supplementary Table S1), these findings provide preliminary evidence for future exploration of BFCS‐cognition relationships.

## 4 Discussion

This study sought to elucidate the relationship between the BFCS, the integrity of its associated white matter tracts, and cognitive function in patients with TLE, with particular emphasis on the impact of HS. The principal findings are as follows: (1) Compared to healthy controls, TLE patients exhibited deficits in global cognition and visual‐spatial memory, with HS potentially exacerbating the severity of these cognitive impairments. (2) In comparison to healthy controls, patients with TLE demonstrated disruptions in BFCS‐related fiber tracts, with individuals suffering from LTLE exhibiting more extensive and pronounced damage than those with RTLE. HS may also contribute to alterations in BFCS‐related fiber tracts in TLE patients. (3) Furthermore, BFCS‐related fiber tracts demonstrated nominal associations with global cognitive function in TLE, with the observed relationship between the Ch4 nucleus and its corresponding tracts being particularly noteworthy. These findings imply a potential involvement of the BFCS in cognitive processing in TLE, suggesting a putative linkage between the Ch4 nucleus and cognitive outcomes that warrants further investigation.

### 4.1 Correlation Between BFCS and Cognitive Function

This study observed that white matter abnormalities in the BFCS of TLE patients show a potential association with cognitive decline, which may represent downstream effects of epileptogenic processes rather than direct causal mechanisms. As the central cholinergic regulatory hub, the BFCS represents the largest aggregation of cholinergic neurons in the brain and plays a pivotal role in cognitive processes (Mufson et al. 2003). This system comprises key nuclei, including the MS, DBB, NBM, preoptic regions, and ventral pallidum (Ferreira‐Vieira et al. 2016). In primates, the BFCS is anatomically divided into Ch1 (MS), Ch2 (vDBB), Ch3 (hDBB), and Ch4 (NBM) subdivisions, which project extensively to cortical and subcortical regions (Knox and Keller 2016, Newman et al. 2012), modulating attention, memory, learning, and executive functions (Schumacher et al. 2021). The BFCS is critically involved in memory consolidation and retrieval, with distinct subdivisions contributing to diverse cognitive domains (Teles‐Grilo Ruivo and Mellor 2013; Wilkins et al. 2020; Ananth et al. 2023). For instance, cholinergic signaling enhances theta oscillations in the hippocampus to facilitate memory formation (Mineur et al. 2022; Wilson and Fadel 2017; Buzsáki 2002), while amygdala connections regulate emotional processing (Hasselmo 2006; Lee et al. 1994). These multimodal regulatory properties explain the broad cognitive consequences of BFCS pathway disruption and highlight its potential as a therapeutic target for cognitive impairment.

Notably, the integrity of Ch4 (NBM) fiber connections emerged as the strongest predictor of cognitive performance. As the primary source of cortical cholinergic input, the Ch4 nucleus governs memory, attention, and motor plasticity (Oswal et al. 2021). Ch4 degeneration has been implicated in memory deficits across neurodegenerative disorders, including AD and PD (Pereira et al. 2020; Zeng et al. 2022; Kilimann et al. 2014; Jiang et al. 2016), a finding corroborated in this study. We propose that white matter damage in TLE disrupts BFCS connectivity, impairing neural signal transmission and network integration, thereby reducing cognitive efficiency. Furthermore, emerging evidence suggests that these structural disruptions may manifest as aberrant large‐scale brain dynamics, as reflected by altered propagation patterns of neuronal avalanches in TLE, which are correlated with memory performance (Duma et al. 2023). Importantly, while our findings establish robust structure‐function correlations, the observational design cannot determine whether BFCS damage directly causes cognitive impairment or constitutes a secondary consequence of broader epileptic network dysfunction. This mechanism not only identifies BFCS as a biomarker for TLE‐related cognitive impairment but also suggests that cholinergic modulation may offer therapeutic benefits.

Intriguingly, despite more extensive BFCS network damage in left TLE patients compared to right TLE counterparts, no significant intergroup cognitive differences were observed. This may reflect compensatory neuroplasticity or limitations in conventional cognitive assessment tools to detect subtle deficits. Future multimodal longitudinal studies with high‐sensitivity evaluations are needed to elucidate structure‐function dynamics and inform precision interventions.

### 4.2 Hippocampal Sclerosis Exacerbates Cognitive Impairment in TLE

This study demonstrates the detrimental effects of hippocampal sclerosis on cognitive function in TLE. Patients with HS exhibited more severe global cognitive deficits, particularly in visual‐spatial memory, compared to non‐HS counterparts. The hippocampus, a critical hub for spatial learning and episodic memory, relies on cholinergic activity for memory encoding and retrieval (Janak and Tye 2015; Wilkins et al. 2020). Its anterior and posterior segments differentially mediate verbal and visuospatial memory, respectively (Reppert et al. 2023). HS, a hallmark of refractory TLE, involves neuronal degeneration in CA1, CA3, and the dentate gyrus, directly impairing hippocampal information integration (Postma et al. 2020; Witt et al. 2015). Left hippocampal head atrophy correlates with verbal learning deficits, while bilateral posterior hippocampal volume loss exacerbates visual‐spatial memory dysfunction, aligning with our findings.

TLE with HS also displayed unique cholinergic pathway damage, particularly in BFCS‐amygdala projections, with left Ch4 to ipsilateral amygdala/hippocampus connectivity showing the most severe disruption. Hippocampal sclerosis likely impairs cognition through dual mechanisms involving both structural and neurochemical pathways. Progressive hippocampal atrophy directly compromises memory processing capacity, while concurrent cholinergic dysregulation disrupts acetylcholine‐mediated modulation of hippocampal‐amygdala networks. Acetylcholine derived from the BFCS, transmitted to hippocampal pathways originating from the MS and vDBB, plays an essential role in governing memory encoding and retrieval processes, as evidenced by prior mechanistic studies (Dutar et al. 1995; Teles‐Grilo Ruivo and Mellor 2013). Disrupted cholinergic signaling exacerbates structural and neurochemical imbalances, creating a vicious cycle. Combined therapies targeting HS and cholinergic circuit repair may disrupt this pathological cascade. Future studies should delineate spatiotemporal interactions between HS and cholinergic dysfunction to guide precision treatment.

### 4.3 BFCS Volume and Connectivity Alterations in TLE

Although no significant group‐level differences in BFCS volume were identified, this observation aligns with the divergent neuropathological mechanisms distinguishing TLE from AD. Unlike AD, where early basal forebrain atrophy predominates, TLE selectively affects hippocampal‐amygdala circuits, indirectly impairing BFCS function through distal network dysregulation rather than direct volumetric loss (Liu et al. 2023; Ballinger et al. 2016). Additionally, BFCS volume changes may require longer disease duration to manifest, and the relatively small sample size may limit detection sensitivity. Future large‐scale longitudinal studies are warranted.

Furthermore, our study observed asymmetric alterations in BFCS‐hippocampal connectivity between LTLE and RTLE patients. Specifically, LTLE patients exhibited extensive bilateral hippocampal connectivity damage, whereas right TLE patients showed impairments predominantly in left hippocampal connections. We hypothesize this asymmetry may reflect lateralized epileptic network propagation and functional reorganization mechanisms. Supporting this view, prior research demonstrates more severe disruption of splenial/hippocampal commissural fibers in left TLE and greater whole‐brain network reorganization in left TLE through dynamic network analysis (Concha et al. 2012; Lopez et al. 2024). The left hippocampus, serving as the verbal memory epicenter, demonstrates greater susceptibility to contralateral epileptic spread via transhemispheric connections. Conversely, right hippocampal damage may be compensated by left prefrontal mechanisms for spatial memory, potentially explaining the attenuated between‐group differences observed in our cohort (Lopez et al. 2024).

Notably, left TLE patients displayed marked bilateral damage to Ch4‐derived fibers targeting the hippocampus and amygdala, indicative of axonal degeneration and microstructural disorganization. This pattern shares features with AD continuum pathology, where hyperconnectivity between right Ch4 and bilateral amygdala correlates with cognitive decline (Zeng et al. 2022). Cholinergic compensatory remodeling in early AD predicts preclinical progression, whereas Ch4 nucleus degeneration in TLE drives cognitive decline through dual mechanisms: disrupted amygdala‐hippocampal integration and maladaptive network compensations. These findings collectively identify Ch4 integrity as a robust predictive biomarker of neurocognitive trajectories and support the development of cholinergic‐targeted therapeutic strategies to ameliorate epilepsy‐associated cognitive deterioration.

## 5 Limitations

First and foremost, the observed correlations suggest a potential role for BFCS alterations in cognitive impairment; these associations may reflect downstream effects of the disease rather than causal mechanisms. This study is subject to several limitations. Firstly, the investigation concentrated predominantly on BFCS‐related fiber tracts within key nuclei associated with cognition, without encompassing the entire brain network. This approach may lead to an incomplete mapping of the cognitive network. Future research endeavors should strive to incorporate whole‐brain analyses. Secondly, the small size of certain BFCS subregions necessitated the aggregation of several smaller subregions for analysis, thereby constraining the capacity to assess the individual contributions of each subregion to cognitive processes. Future research should explore the distinct functions of each subregion in greater detail. Third, the significant disparity in educational attainment between the patient cohort and healthy controls in this study may introduce selection bias. Educational disparities, along with unmodeled clinical confounders encompassing antiepileptic drug effects, disease duration, and seizure frequency, necessitate sensitivity analyses integrated with multimodal designs to strengthen causal inference validity. Furthermore, the methodology employed to evaluate BFCS fiber tract damage in patients with TLE relied on DTI parameters, which may be considered relatively simplistic. Integrating multiple imaging modalities, such as fMRI, could yield more comprehensive and reliable findings. Lastly, our study evaluated BFCS fiber damage exclusively through indirect diffusion metrics such as FA and MD without employing direct connectivity quantifiers like fiber‐specific weights or lengths. These localized measures fail to capture topological network roles within whole‐brain systems. Future research should adopt network‐level approaches to better delineate BFCS functional roles in neural circuitry.

## 6 Conclusion

Our study identified a correlation between cognitive impairment in patients with TLE and damage to projective fibers associated with the BFCS, with hippocampal sclerosis emerging as a significant factor exacerbating this fiber damage. Notably, the Ch4 subregion exhibited a potential correlation between its projected fiber connections to the hippocampus and amygdala and cognitive performance, suggesting that both the BFCS and the Ch4 subregion may play a role in the cognitive deficits observed in TLE. These findings suggest that future therapeutic strategies might focus on maintaining or restoring the BFCS and its connections, particularly within the Ch4 subregion, to potentially mitigate cognitive decline and improve outcomes in TLE patients. Further research could explore neuroprotective agents or rehabilitative therapies aimed at supporting the cholinergic system and enhancing cognitive function in TLE patients.

## Author Contributions

**Beibei Shen**: conceptualization, data analysis, writing–original draft, writing–review and editing. **Lina Jiang**: data analysis, writing–review and editing. **Yi Shi**: data analysis, writing–review and editing. **Jiahui Guo**: data analysis and figure modification. **Bofei Chen**: data analysis and manuscript revision. **Zhiruo Qiu**: data analysis, manuscript revision and figure modification. **Shuang Wang**: writing–review and editing. **Zhefeng Yuan**: writing–review and editing. **Jiajia Fang**: conceptualization, writing–review and editing.

## Funding

This work was funded by the National Natural Science Foundation of China (grant no. 82401698), the major science and technology projects of Zhejiang province (grant no. 2023C03080), and the Medical Interdisciplinary Innovation Program 2024, Zhejiang University School of Medicine.

## Ethics Statement

This study was approved by the Ethics Committee of the Second Affiliated Hospital, Zhejiang University School of Medicine (approval no. 2014151). Informed consent was obtained from all participants, and the research adheres to all applicable ethical standards.

## Conflicts of Interest

The authors declare no conflicts of interest.

## Supporting information




**Supplementary Materials**: brb371090‐sup‐0001‐TableS1.xlsx

## Data Availability

The data supporting the findings of this study are available upon reasonable request from the corresponding author.
